# Mechanisms Involving Myocardial Injury in Tropical Stings and Bites

**DOI:** 10.1155/2017/4960505

**Published:** 2017-11-02

**Authors:** Thirunavukarasu Kumanan, Mahesan Guruparan, Ratnasamy Vithiya, Indika Gawarammana

**Affiliations:** ^1^University Medical Unit, Teaching Hospital Jaffna, Jaffna, Sri Lanka; ^2^Department of Cardiology, Teaching Hospital Jaffna, Jaffna, Sri Lanka; ^3^Faculty of Medicine, University of Peradeniya, Kandy, Sri Lanka

## Abstract

It is known that a number of toxic substances produce myocardial injury by several mechanisms involving interruption of coronary blood flow due to stimulation of clotting mechanism and coronary vasospasm. Number of toxic substances may cause direct myocardial toxicity independent of coronary blood flow. Acute myocardial injury due to stings and bites is a rare entity and not well understood. Here we illustrate a case of myocardial injury due to Russell's viper envenomation.

## 1. Introduction

Bites and stings are commonly encountered clinical problems in the tropics. Toxins due to these stings and bites could cause a wide array of clinical manifestations. Myocardial injury is a relatively rare entity of bites and stings and is increasingly recognized in the developing tropical nations due to relatively easier access to cardiac biomarkers and imaging in day to day practice. Several models were proposed over time to explain the mechanism of myocardial injury. However single reports of toxic myocarditis lack detailed histologic characterization and clear association with the offending agent to explain the exact pathogenesis [1].

## 2. Case History

A 17-year-old boy presented to the peripheral hospital soon after Russell's viper bite. Bilateral ptosis was noted on admission and he was treated with ten vials of Indian polyvalent antivenom serum (AVS). Persistent coagulopathy evident after 6 hours by the prolonged whole blood clotting time (WBCT) resulted in the administration of further ten vials of AVS. With the impending respiratory failure he needed endotracheal intubation and was transferred to the emergency unit of Teaching Hospital Jaffna for ventilator support. In spite of significant coagulopathy his haemodynamic status was reasonably stable during the next 10 hours. He was treated with fresh frozen plasma and ten more vials of AVS subsequently.

His initial investigations revealed neutrophil leucocytosis, CPK of 2,755 U/l (52–336 U/l), and a serum creatinine of 224 micromol/l. The onset of circulatory compromise 12 hours later was indicated by the tachycardia and hypotension which was refractory to fluid resuscitation, and the necessity for ionotropic support prompted us to think about the possibility of cardiac toxicity. Two-dimensional echogram was performed which revealed global hypokinesia and severe left ventricular dysfunction with an ejection fraction of 30%. His cardiac troponin I level was 10.3 ng/ml (0.015–0.15 ng/ml). Despite intensive supportive care, he succumbed to myocardial damage within 48 hours of envenomation.

## 3. Discussion

Insufficient blood supply to the myocardium can result in myocardial ischemia, injury, or infarction, or all three. ECG is a readily available noninvasive investigation that is very useful in picking up myocardial damage. Currently, troponin is the gold standard biomarker for myocardial injury and is used commonly in conjunction with creatine kinase-MB (CK-MB) and myoglobin to enable a more rapid diagnosis of such events [2].

In a patient with tropical bite or sting, unexplained tachycardia, hypotension, or hypoxaemia in the absence of a respiratory cause would raise the suspicion of myocardial involvement. An ECG with diffuse ST-T changes would prompt the treating clinician to rule out myocardial damage by a cardiac biomarker.

Numbers of toxic substances produce myocardial injury independent of interruption in coronary blood flow. It includes biogenic amines and drugs like anthracyclines. Catecholamines induced cardiotoxicity is characterized by damaged muscle cells with myofibrillar hypercontraction, contraction bands, and mitochondrial calcium deposition. Catecholamine induced myocardial damage progresses as the demand of oxygen increases with increased metabolic rate by stimulation of catecholamine receptors and a mismatch occurs in myocardial perfusion [3]. Anthracyclines like daunorubicin and adriamycin can cause similar acute myocardial damage and delayed response to cumulative effects of these drugs results in interstitial fibrosis and myocardial degeneration [4].

Venom and toxins contain variety of organic substances that cause devastating clinical manifestations. Stings from insects of order Hymenoptera (honeybee, wasps, and ants) are frequent events in Northern Sri Lanka. The venom contains a mixture of peptides and enzymes that cause a nonallergic or allergic local reaction, anaphylaxis, or a systemic toxic reaction such as oedema, vomiting, and seizure. Unusual reactions include cardiac ischemia, cerebral infarction, and encephalomyelitis [5].

Stings of white scorpion* (Hottentotta tamulus)* were not recorded in Sri Lanka until 1990, the species said to have migrated to Jaffna peninsula with the movement of Indian peace mission. There has been a gradual increase in cases reported with* Hottentotta tamulus *stings since the end of civil war in 2009 with confirmed 22 hospital admissions (out of 78 stings by scorpions) in 2013 [6].

Scorpion venom contains a mixture of several low molecular weight basic proteins, neurotoxins, nucleotides, amino acids, oligopeptides, cardiotoxins, nephrotoxins, haemolytic toxins, phosphodiesterase, phospholipase A, hyaluronidase, acetylcholinesterase, glycosaminoglycans, histamine, serotonin, 5-hydroxyptamine and proteins that inhibit protease, angiotensinase, succinate-dehydrogenase, ribonuclease, and 5-nucleotidase. Multiple toxins may be present in the venom of a single species which can produce a synergic effect in the victim [7].

Toxins in Sri Lankan white scorpion venom are not studied yet; however two mechanisms are thought to contribute to its cardiotoxic properties. The direct cardiotoxic effect of the venom causing toxic myocarditis by reduction of Na-K-ATPase and catecholamine induced myocarditis by releasing adrenaline and noradrenaline from neurons, ganglia, and adrenals. The other mechanism is myocardial ischemia caused by coronary vasospasm due to release of vasoactive, inflammatory, and thrombogenic peptides and amine constituents (histamine, serotonin, bradykinin, leukotrienes, and thromboxane) [8]. This acts on the coronary vasculature and induces coronary vasospasm and facilitates platelet aggregation, leading to thrombosis [9]. Bites due to* Daboia russelli* (Russell's viper) is common in Northern Sri Lanka. Cardiac toxicity following Sri Lankan viper bites does not seem to be a common feature though it is common following Burmese and European vipers. Seneviratne et al. [10] in a study concluded that cardiac symptoms were present in number of victims of envenomation and transient ECG changes were noted in only 2 patients out of 45 patients studied during the study period. However troponin I levels were not elevated in any of them. The mechanism by which venom causes cardiotoxicity is still not clearly understood. The proposed mechanisms are direct cardiotoxic effects causing myocardial damage, disseminated intravascular coagulation leading to coronary vessel thrombosis, and coronary vasospasm caused by safratoxins [11–15]. It is believed most cardiotoxins from snakes cause myocardial degeneration by acting on the extracellular surface to increase the cytosolic calcium ions and subsequently leading to calcium dependent noncytosolic system activation [16]. In 1996 Dissanayake and Sellahewa described an anterolateral myocardial infarction following Russell's viper in a 47-year-old man [17]. In 2012 Silva et al. [18] have reported an inferior ST elevation myocardial infarction following direct intravenous envenomation by Russell's viper in a 33-year-old male. Based on the clinical findings and the investigations it is apparent that the bee sting and the scorpion sting have resulted in significant coronary vasospasm and Russell's viper envenomation has activated the coagulation cascade and hinders the coronary blood flow. Both mechanisms played a pivotal role in myocardial injury in these cases.

In conclusion a wide array of stings and bites occur in the tropics in day to day life and myocardial toxicity is increasingly recognized as a potentially fatal complication of these events. The treating clinician should be vigilant enough to act on this devastating complication. Early recognition and prompt intervention would save hundreds of lives due to this dreaded complication.

## Abbreviations

AVS: Antivenom serum
CPK: Creatine phosphokinase
ECG: Electrocardiogram
WBCT: Whole blood clotting time.
